# Factors influencing patients' opt-in intention of exchanging health information

**DOI:** 10.3389/fpubh.2022.907141

**Published:** 2022-10-17

**Authors:** Xijing Zhang, Runtong Zhang

**Affiliations:** Department of Information Management, School of Economics and Management, Beijing Jiaotong University, Beijing, China

**Keywords:** opt-in intention, health information exchange, the theory of planned behavior, health status, transparency

## Abstract

**Introduction:**

Health information exchange (HIE) exhibits tremendous benefits in improving the quality of healthcare and reducing healthcare costs. However, it also poses challenges related to data security, data privacy, patient engagement, etc.

**Objective:**

This study aimed to explore the factors affecting patients' opt-in intention to HIE by using an empirical study based on the theory of planned behavior.

**Methods:**

A Web-based survey was conducted involving 501 valid participants in China (69% validity rate).

**Results:**

Information sensitivity and perceived HIE transparency affected the patients' opt-in intention to HIE through the mediation of perceived behavior control and trust in HIE. Information sensitivity negatively influenced perceived behavior control (−0.551, *P* < 0.001) and trust in HIE (−0.489, *P* < 0.001). Perceived transparency of HIE positively influenced perceived behavior control (0.396, *P* < 0.001) and trust in HIE (0.471, *P* < 0.001). Moreover, patients' opt-in intention to HIE can be positively affected by perceived HIE transparency (0.195, *P* < 0.001) and trust in HIE (0.294, *P* < 0.001). In addition, the moderating effect of health status was positive and significant between trust in HIE and opt-in intention to HIE but not between the perceived behavior control and opt-in intention to HIE.

**Conclusion:**

This study contributes to the theory of planned behavior and enriches the literature on HIE efforts. HIE administrators should design personalized health services on the basis of these different health statuses to successfully achieve patients' opt-in intention to HIE.

## Introduction

Health information exchange (HIE) is the sharing of electronic health information among different medical professionals and medical institutions with the help of health information technology (1). When patients directly switch from one medical institution to another, timely sharing of key patient information can prevent readmission, improve diagnosis rate, reduce repeated examinations, and avoid medication mistakes (2–5). However, some obstacles hinder the development of HIE. For example, researchers can retrieve electronic databases to reuse the data; therefore, seeking informed consent from patients when obtaining data is unrealistic for each platform (6). As important stakeholders of HIE projects, patients can influence data collection and information sharing (7). Opt-in intention to HIE is the extent to which a patient is willing to rely on HIE as a useful and reliable technology to be used by healthcare entities for information dissemination (8).

The theory of planned behaviors (TPB) is a social psychological theory explaining the relationship between attitude and behavior. As one of the important theories to predict and explain human behavior, TPB is widely used in healthcare, management, education, psychology, information science, economics, and other fields (9–11). Based on TPB, perceived behavior control, attitude, and subjective norms are three factors affecting opt-in intention (12). However, in the healthcare industry, Deng et al. (13) and Heart et al. (14) found that subjective norms will not significantly affect the patients' intention to use health information and communication technology. The subjective norms depend on normative beliefs. This normative belief has two meanings given as follows: one is the degree to which individuals perceive that their significant people expect them to perform certain behaviors; another is the extent to which individuals conform to these views. In line with these meanings, social pressure is difficult to understand directly by obeying or disobeying the wishes of others (15). Therefore, subjective norms do not have a good effect in reflecting the influence of social pressure on individual behavior. Moreover, attitude is a broad concept and cannot be described using some single words. Studies using a single measurement to decide attitudes, such as interesting–boring, useful–useless, upset level, and regrets, are available (12, 16). Many scholars use trust to represent attitude when studying the patients' opt-in intention to HIE (8, 17). Thus, we reset the TPB model by removing subjective norms and further expand the factor attitude to trust.

Thus, according to TPB, perceived behavior control and attitude (i.e., trust) are the two factors affecting opt-in intention. Perceived behavior control is widely applied to behavior research models, referring to the degree to which individuals feel that they can control or master a particular behavior (18). Trust is defined as trusting beliefs, which are the cognitive beliefs shaped by the trustor (19). Meanwhile, information sensitivity refers to the degree to which individuals pay attention to the information in a particular environment (20) and is closely linked to privacy concerns. Information sensitivity and privacy concerns are major barriers to patients' acceptance of information sharing. Moreover, transparency refers to the right to know what type of health information is shared, with whom, when, and for what purposes (17) and represents how patients can understand the type of information shared, frequency, senders, recipients, and purpose of exchange. The transparency of privacy policy dimensions is a sound rationale for consumers to trust in HIE competence (17). Thus, information sensitivity and HIE transparency may be factors affecting perceived behavior control and trust in HIE.

This study is conducted to investigate how individual consumers develop their opt-in intention to HIE by utilizing the TPB and advances in this research area. An important knowledge gap is also addressed by applying this model to assess the factors affecting patients' opt-in intention to HIE. Previous studies have found physicians' influence of HIE (21, 22), but limited empirical studies have examined the effect of health status on individuals' intentions to disclose their personal information in the field of HIE [e.g., Esmaeilzadeh's study (23) and Yaraghi's study (24)]. Moreover, no prior work has explored the moderating effect of health status on the relationship between perceived behavior control and patients' opt-in intention to HIE and between trust in HIE and patients' opt-in intention to HIE. Thus, how the health status strengthens or suppresses the effects of perceived behavior control and trust in HIE on opt-in intention to HIE is a valuable research field (22). Especially, research on HIE in China is still in its infancy because foreign research results cannot be directly applied in this country (25, 26).

## Hypotheses

The research model describes how patients' opt-in intention to HIE is influenced. The independent variables are information sensitivity and perceived transparency of HIE, the mediators are perceived behavior control and trust in HIE, the dependent variable is opt-in intention to HIE, and the moderate variable is health status.

Whether consumers are willing to disclose personal information is related to the sensitivity of such information (27). Patients may intend to hide health information from healthcare providers if their needs for protecting information sensitivity are not met. This behavior occurs because the low sensitivity of the information required by the website will reduce users' anxiety about personal information being leaked or shared, thereby making them feel that they have high control over personal information (28).

The content of privacy statements is founded as a significant factor to predict consumer trust in many industries (29–31). If health records are exchanged confidentially, patients will increase trust toward HIE (32). Consumers' responses to privacy issues depend on the type of health information that is exchanged electronically with other healthcare providers (33). A trustful attitude toward an HIE system is a result of a solid match between the HIE mechanisms and security or privacy requirements (34). The greater the sensitivity involved in medical information, the more serious people's privacy concerns will be when releasing it, which will reduce their trust toward behaviors (35). This discussion results in the following hypotheses:

H1: Information sensitivity has a negative impact on perceived behavior control.
H2: Information sensitivity has a negative impact on trust in HIE.

If the privacy policy is transparent, patients can recognize the suppliers, information type, and the switching mechanism (12). Consequently, patients will have a comprehensive understanding of main functions of the HIE and related sharing procedures and safety mechanisms. Then, patients will perceive that the electronic exchange of information among healthcare providers is a convenient and cost-effective sharing method (36). Accordingly, people will feel that they can control the process of HIE, and the perceived behavior control can be improved.

If individuals realize that some technology is logically reliable, they will become more likely to rely on it emotionally (30). Clearly defining and promoting privacy policies are efficient and practical methods for patients to realize that information is shared electronically between medical institutions and individuals in a complete and unblemished way. This recognition may increase the trust in HIE capabilities and encourage patients to believe that the HIE technology is a true expert system in the field of information sharing. In other words, patients believe that HIE is trustworthy because it has the necessary technical basis and effective communication mechanism, which can effectively share health information between providers. Thus, we propose the following hypotheses:

H3: Transparency of HIE has a positive impact on perceived behavior control.
H4: Transparency of HIE has a positive impact on trust in HIE.

According to the TPB, perceived behavior control and attitude (i.e., trust) in HIE are the two main factors affecting opt-in intention to HIE (12). Users believe that if they have enough information (e.g., website privacy settings and legal requirements) to ensure that the information they publish is safe, then they will be willing to share their personal medical information. Simultaneously, if they think they have the skills and tools to deal with the consequences of releasing the information, they will be happy to allow information exchange among medical instructions.

Mital et al. (37) argued that trust promotes individuals' willingness to share information. Given that patients often cannot adopt HIE directly, they can develop attitudes, beliefs, and emotions that participate in the concept of shared efforts. Therefore, the use of perceptual measures should be evaluated, rather than the actual selection of the behavior. The patients' sense of security and strong comfort in relying on the HIE network can increase the intention to opt-in the HIE system. The sense of trust becomes their tool to help them decide to support medical institutions in using HIE to improve the quality of care and reduce the cost of care. Accordingly, perceived behavior control and trust in HIE can encourage patients to share their medical records with related entities. Thus, we hypothesize as follows:

H5: Perceived behavior control has a positive impact on opt-in intention to HIE.
H6: Trust in HIE has a positive impact on opt-in intention to HIE.

Individuals will have different views on information disclosure under different health conditions (38). The extent to which perceived behavior has control on opt-in decisions may vary depending on the current health status of people. People who are always plagued by illness are more anxious about HIE behavior held by healthcare providers than healthy people because they have more privacy concerns and feel less control over their private health information (39). However, patients with poorer health status are more likely to choose HIE for better therapeutic effects, regardless of the level of perceived behavior control (39). Given the physical or mental weaknesses, they believe their control over HIE is limited, but they may allow medical institutions to exchange their electronic information in the hope of helping suppliers access their complete and updated medical records. Thus, health status enhances the relationship between perceived behavior control and opt-in intention to HIE.

The psychological characteristics of patients are anxiety, depression, and pessimism (40). Patients with worse health status have better information needs than other patients and will rely more on HIE to improve health statuses and relieve mental stress. Accordingly, patients with poorer health status have a stronger sense of and dependence on the HIE than others for the purpose of obtaining social support and care whether they trust in HIE or not. Health status can especially strengthen the effect of trust in HIE on patients' intention to HIE. On the contrary, people with healthy status have less demands for the HIE system. Whether they trust in HIE or not and perceive that they can control the process of HIE or not, they will not have strong intention to use it, indicating that the relationship between perceived behavior control and opt-in intention to HIE and between trust in HIE and opt-in intention to HIE can be weakened when health status is well. Thus, we hypothesize as follows:

H5m: Health status positively moderates the relationship between perceived behavior control and opt-in intention to HIE.
H6m: Health status positively moderates the relationship between trust in HIE and opt-in intention to HIE.

## Materials and methods

### Measurement development

The constructs included in the model were measured in accordance with the literature. The variables in the research model were measured using a five-point Likert response format, ranging from strongly disagree to strongly agree (as shown in Supplementary material), which was validated by previous works. Items measuring opt-in intention to HIE were modified from the studies by Venkatesh et al. (41) and Angst et al. (42). A total of five items reflecting perceived health information sensitivity and three items reflecting health status were adapted from the scales by Bansal and Gefen (35). We adapted the method mentioned in studies by Chua et al. (43) to measure the transparency of HIE. Trust in the HIE was measured by modifying the methods in the studies conducted by Wu et al. (30). Perceived behavior control was measured by modifying the methods in the studies by Hsieh (25).

### Analysis tool selection

Structural equation modeling (SEM) is useful in analyzing the causal relationships between research models and for exploratory research and theory development. This study adopted the partial least squares (PLS)-SEM method to analyze the research model and used SmartPLS version 3.0 to estimate path models with latent variables and their relationships.

### Data collection and respondents

The formal investigation was conducted in March 2020 in a Web-based platform. The study was approved by the Ethical Committee of the School of Economics and Management, Beijing Jiaotong University. At the beginning of the online survey, the HIE technology was described in detail to the respondents to ensure that they fully understood the background and purpose of the research as most respondents were not aware of HIE (44). We specified an additional qualification that they must know HIE by defining screening questions to check whether their experience in the HIE project satisfied our criteria. They were asked to describe whether and why they were familiar with HIEs before answering the main survey questions. The completion time was also checked; if the completion time was obviously lower than the average time, the survey would be invalid. In total, 728 individuals attempted the survey, and 69% (501/728) of them were aware of HIE by visiting a healthcare provider participating in an HIE project or attending HIE project before. Others were not aware of HIE or were aware of HIE because of other reasons, such as reading newspapers, through Internet searching, families, and friends.

Table 1 shows the demographics of the sample. The demographic characteristics of the participants showed that the age of respondents ranged from 18 to 65 years. Approximately 70% of respondents were 20–39 years old, and only 2% were 60 years and older. Approximately 45% of the participants had education of below high school, and some had college degrees. Close to 46% had bachelor's degrees, and the remaining had graduate degrees (master's degree or doctorate). The largest number of participants (68%) lived in urban areas. More female (52.3%) than male (47.7%) participants were involved. A previous study has also reported that HIE users are likely to be young, female, and educated (17). Therefore, the sample met our requirements.

**Table 1:** Sample demographics (*N* = 501).

**Demographic characteristics**	**Participants, *n* (%)**
**Age (years)**
< 20	8 (1.6)
20–29	127 (25.35)
30–39	222 (44.31)
40–49	112 (22.36)
50–59	31 (6.19)
60 and above	1 (0.02)
**Gender**
Male	239 (47.7)
Female	262 (52.3)
**Resident status**
Urban	341 (68.06)
Rural	160 (31.94)
**Education**
Junior middle school or below	3 (0.60)
High school	65 (12.97)
Junior college	158 (31.54)
Bachelor's degree	225 (46.31)
Master's degree	43 (8.58)
Doctor's degree	7 (1.40)

## Results

### Hypothesis testing

We first calculated the outer loading for all construct by using SmartPLS software 3.0, and all values were > 0.700. The Cronbach alpha of each construct was greater than the cutoff value of 0.700 (45), which indicated good reliability of scales. The convergent validity of scales was acceptable because the composite reliability (CR) and average variance extracted (AVE) of constructs exceeded the cutoff values of 0.700 and 0.500, respectively. All the diagonal values were >0.7 and exceeded the correlations between any pair of constructs; thus, the discriminant validity was acceptable.

We added gender, age, living area, and education level as control variables. Cohen *f*^2^ was used to assess the effects of the control variables (46). In the regression relationship, all the variables had good explanations because all the multivariate coefficients of determination (*R*^2^) were higher than 0.67. We contended that all control variables had insignificant effects on the research model (i.e., insignificant: <0.020; small: ≥0.020 and <0.150; medium: ≥0.150 and <0.350; and large: ≥0.350).

**Figure 1:**
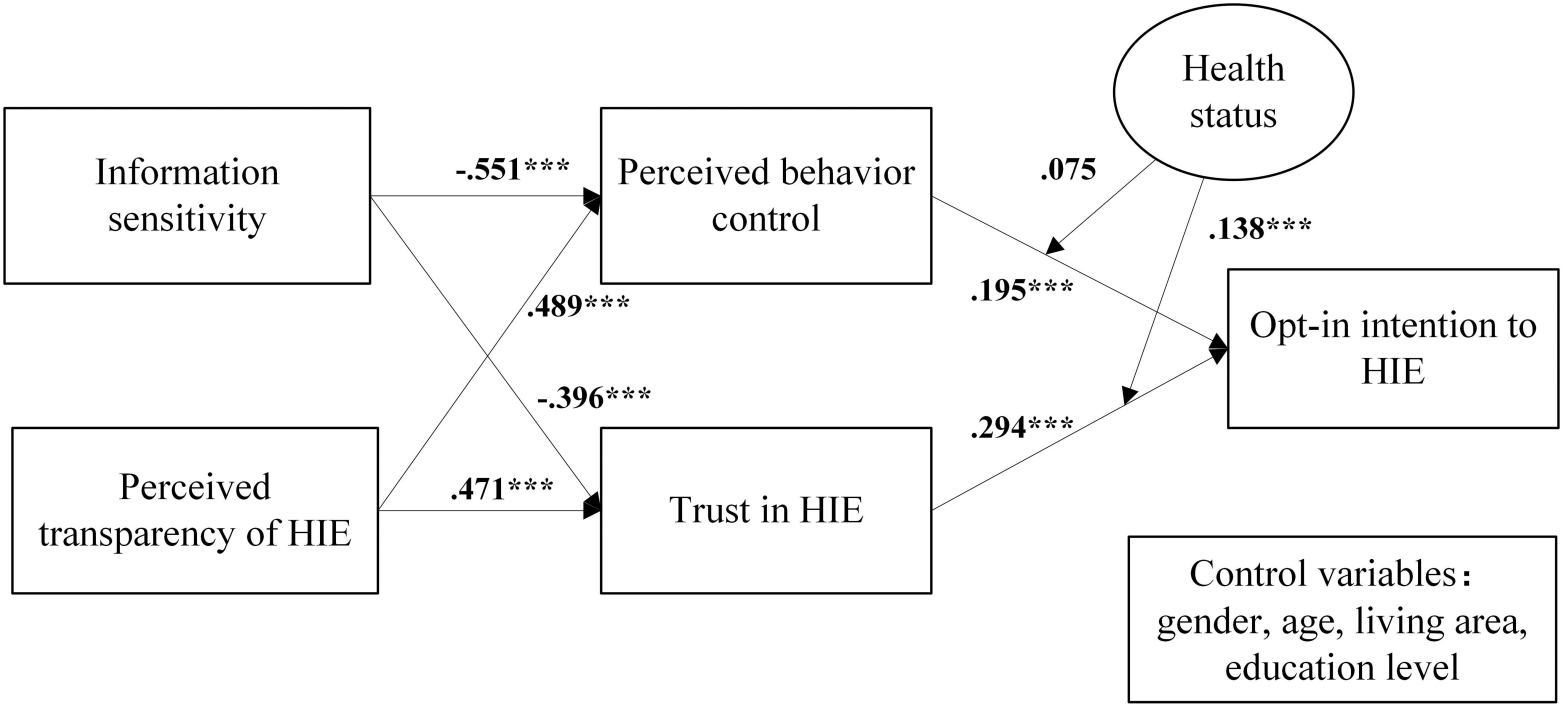
Research model with path coefficients. ***P < 0.001.

According to Figure 1 and Table 2, all hypotheses were supported (i.e., H1, H2, H3, H4, H5, H6, and H6m), except H5m. The possible reasons for the insignificant relationship are stated in the next section, and we estimated the medicating effect by bootstrapping. Table 3 shows the effect size of each construct in the research model. The results indicate that information sensitivity and perceived transparency of HIE had medium influences on the perceived behavior control and trust in HIE, and the influences of perceived behavior control and trust in HIE on opt-in intention to HIE were weak with small effect sizes.

**Table 2:** Hypothesis testing.

**Hypothesis**	**Path coefficient**	***T*-test**	***P*-value**
H1: Information sensitivity has a negative impact on perceived behavior control.	−0.551	12.788	< 0.001
H2: Information sensitivity has a negative impact on trust in HIE.	−0.489	12.516	< 0.001
H3: Perceived transparency of HIE has a positive impact on perceived behavior control.	0.396	9.160	< 0.001
H4: Perceived transparency of HIE has a positive impact on trust in HIE.	0.471	12.049	< 0.001
H5: Perceived behavior control has a positive impact on opt-in intention to HIE.	0.195	4.711	< 0.001
H6: Trust in HIE has a positive impact on opt-in intention to HIE.	0.294	5.582	< 0.001

**Table 3:** Partial least squares effect size analysis.

**Variables**	***R*** ^**2**^		**Δ*R*^2^**	**Cohen *f*^2^**	**Effect size**
	**In**	**Out**			
**Opt-in intention to HIE**
Perceived behavior control	0.900	0.894	0.006	0.06	Small
Trust in HIE	0.900	0.888	0.012	0.12	Small
**Perceived behavior control**
Information sensitivity	0.869	0.832	0.037	0.282	Medium
Perceived transparency of HIE	0.869	0.850	0.019	0.145	Medium
**Trust in HIE**
Information sensitivity	0.893	0.863	0.030	0.280	Medium
Perceived transparency of HIE	0.893	0.865	0.028	0.262	Medium

We conducted additional analysis by bootstrapping to confirm the mediating effect, as shown in Table 4. The indirect relationship between information sensitivity and opt-in intention to HIE was significant (*p* < 0.01 and exclusive 0 among confidence intervals), indicating that the mediating effects of perceived behavior control and trust in HIE between information sensitivity and opt-in intention to HIE existed. Similarly, the mediating effects of perceived behavior control and trust in HIE between perceived transparency of HIE and opt-in intention existed because the *p*-value of path was <0.001 and exclusively 0 among confidence intervals. Furthermore, the direct and total effects were all significant, showing that the partial mediating effects existed between information sensitivity and opt-in intention and between perceived transparency of HIE and opt-in intention.

**Table 4:** Path coefficients by the bootstrapping method (*n* = 5000, 95% CI).

**Effects**	**Path coefficient (SD)**	***P*-value**	**Confidence interval**
**Direct effects**
Information sensitivity -> perceived behavior control	−0.551 (0.043)	< 0.001	0.459–0.635
Information sensitivity -> trust in HIE	−0.489 (0.039)	< 0.001	0.413–0.567
Perceived behavior control -> opt-in intention to HIE	0.124 (0.041)	< 0.001	0.044–0.203
Perceived transparency of HIE -> perceived behavior control	0.396 (0.044)	< 0.001	0.313–0.488
Perceived transparency of HIE-> trust in HIE	0.471 (0.039)	< 0.001	0.392–0.548
Trust in HIE -> opt-in intention to HIE	0.187 (0.049)	< 0.001	0.087–0.279
**Indirect effects**
Information sensitivity -> opt-in intention to HIE	−0.160 (0.032)	< 0.001	0.101–0.223
Perceived transparency of HIE -> opt-in intention to HIE	0.138 (0.028)	< 0.001	0.086–0.192
**Total effects**
Information sensitivity -> opt-in intention to HIE	−0.341 (0.055)	< 0.001	0.234–0.451
Perceived transparency of HIE -> opt-in intention to HIE	0.355 (0.047)	< 0.001	0.256–0.444

### Moderating effect

The moderating effect of health status on the paths of trust in HIE to opt-in intention to HIE was significant (β = 0.138, *p* = 0.006), whereas the moderating effect of health status on the paths of perceived behavior control to opt-in intention to HIE was insignificant (β = 0.075, *p* = 0.016). We performed simple slope analysis to further explore the moderating effect of trust in HIE on opt-in intention.

**Figure 2:**
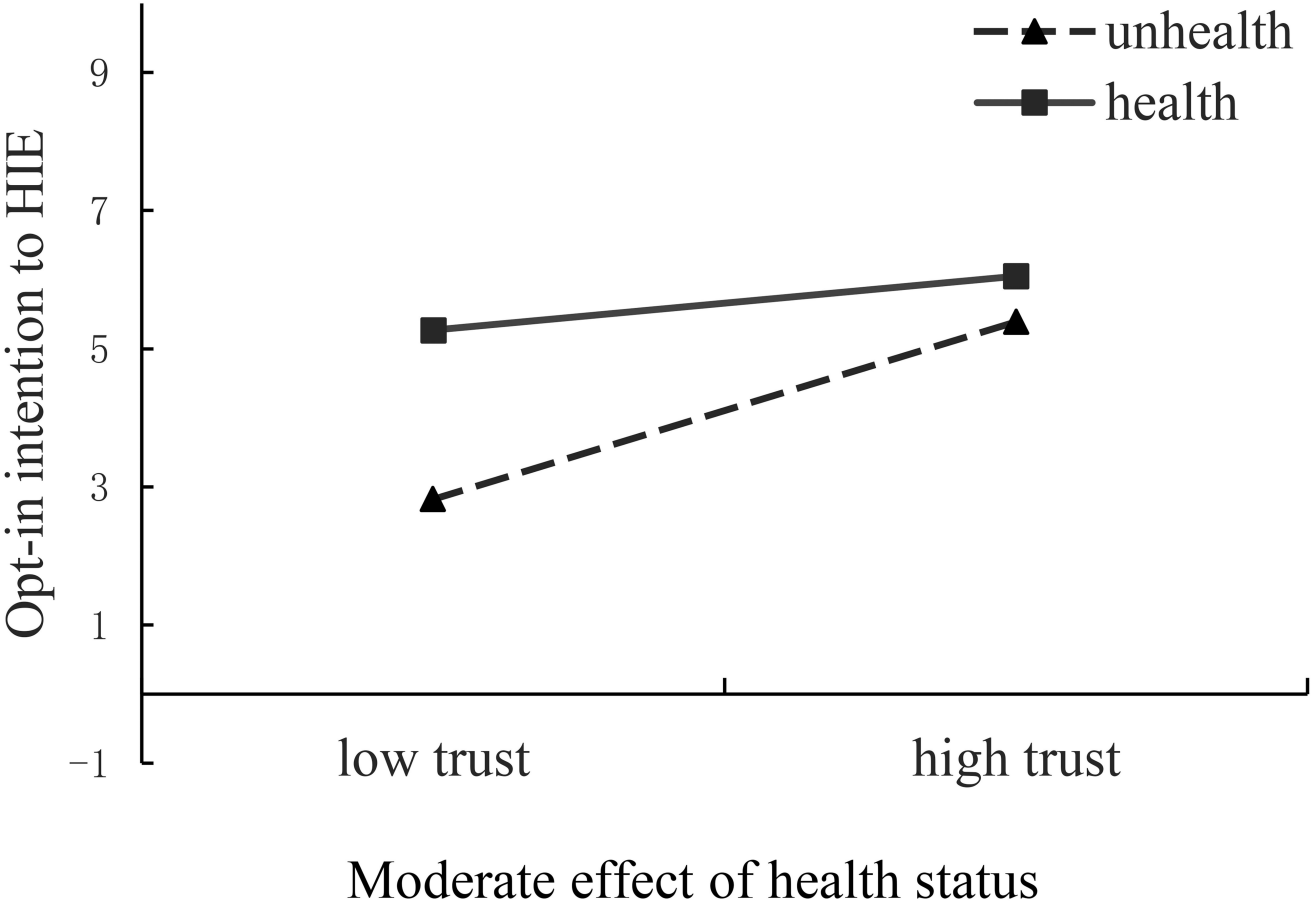
Interaction graphs.

The results in Figure 2 revealed that a significant difference existed in the relationship between trust in HIE and opt-in intention between healthy and unhealthy individuals. This difference implies that individuals who perceive their health status to be worse have a higher level of influence on the relationship between trust in HIE and opt-in intention to HIE than those who perceive themselves as healthy. Thus, unhealthy status has a stronger moderating effect on the relationship between trust in HIE and opt-in intention to HIE than healthy status. Therefore, the results indicate that the linkage of trust in HIE with opt-in intention is positively moderated by the health status.

## Discussion

This study explores the factors affecting opt-in intention to HIE based on the TPB control, and it is one of the first attempts to study the moderating effect of health status on patients' opt-in intention to HIE. Moreover, this study applied the famous TPB widely used in people's behavior intention to HIE context. First, we drew upon the TPB to explain how patients' opt-in intention is affected in the context of HIE. We clarified that the patients' opt-in intention to HIE can be affected by information sensitivity and perceived HIE transparency through the mediation of perceived behavior control and trust in HIE. Information sensitivity and perceived HIE transparency directly impact the perceived behavior control and trust in HIE and indirectly affect the patients' opt-in intention to HIE because all the paths were significant as per hypothesis testing. The path coefficient from trust in HIE (0.294) was significant and higher than that from perceived behavior control (0.195) to opt-in intention to HIE. Therefore, trust in HIE is an important variable in the context of HIE rollout. HIE policymakers should take actions to increase reliability and transparency of sensitive information for fostering patients' trust. For example, hospitals can publicize privacy protection policies to patients in a timely manner, explaining how information is collected and how it is used and stored in an easy-to-understand manner so that patients know that their personal privacy is closely protected. At the same time, the amount and type of required information can be reasonably set, and some highly sensitive information filling requirements can be set as optional so as to avoid forcing patients to provide sensitive personal information, thus cultivating patients' trust in the platform and reducing their concerns about information privacy sharing.

Second, information sensitivity had negative and significant influences on perceived behavior control (−0.551) and trust in HIE (−0.396). The perceived transparency of HIE also significantly affected the perceived behavior control (0.489) and trust in HIE (0.471). Thus, healthcare entities should implement strategies to demonstrate HIE policies for the purpose of generating knowledge of operation in HIE, resolving uncertainty in the process of data sharing, and advancing awareness of HIE. For example, practitioners and government officials can promote the main objectives and policies of HIE by holding national education programs, which are accessible to a wide range of people. The Internet, such as forums of health, is an effective place to broadcast HIE efforts and increase the general public awareness on HIE mechanisms. Moreover, during the outbreak of the new coronavirus, the short board of HIE development was also shown. Given the lack of policy and regulatory guidance, data exchange and sharing were difficult during the COVID-19 pandemic because of concerns about data security and patient privacy. With some supports by economic laws (47, 48), a great deal of assurance can be achieved for the rapid data collection during possible future outbreaks of infectious diseases.

Third, the findings on the moderating effect indicate that the health status may change the beliefs of consumers about the extent to which their health information will be released. When patients are in an unhealthy status, the influence of trust in HIE on opt-in intention to HIE will be enhanced. Given the unexpected results regarding the moderating role of health status between the perceived behavior control and opt-in intention to HIE, further studies can deeply investigate the effect of health status, which may be more complex than we discussed. The HIE administrators must design personalized health services based on these different health demands and especially focus on a non-stigmatizing service design. Accordingly, patients' opt-in intention to HIE will be enhanced.

The limitations of the study must be considered. First, we only focused on the moderating effect of health status on patients' opt-in intention to HIE. Other factors can be added for further investigation, for example, patients' social network. Second, we only considered subjects who are in China. China has a large population and unbalanced healthcare development in different areas. There may be different aspects between China and other countries. Therefore, the generality of the results should be further explored in other countries. Third, this study only collected data through one cross-sectional survey; thus, we cannot capture patients' dynamic changes of attitudes toward all variables. Last, although our sample met the characteristics of typical HIE users, the number of respondents was relatively small considering the feature of Chinese census data.

## Conclusion

The patients' opt-in intention to HIE is crucial for the wide use of HIE. This study clarified that patients' opt-in intention to HIE could be affected by information sensitivity and perceived transparency of HIE through the mediation of perceived behavior control and trust in HIE. The perceived behavior control and trust in HIE were positively and significantly affected by information sensitivity and perceived HIE transparency, respectively. In terms of the moderating effect, health status was confirmed to enhance the relationship between patients' trust in HIE and opt-in intention to HIE. These findings are as follows: (1) Educating consumers about HIE mechanisms and sharing procedures to appeal to their awareness of sensitive information policies and mechanisms of HIE are important because of the difficulties in each confirmation of HIE; (2) researchers, physicians, and policymakers should design personalized health services based on these different health statuses for acquiring patients' opt-in intention to HIE; and (3) enhancing research collaboration and information sharing is necessary to improve the timeliness and effectiveness of clinical trials and enhance the research response capacity to public health emergencies.

## Data availability statement

The raw data supporting the conclusions of this article will be made available by the authors, without undue reservation.

## Ethics statement

The studies involving human participants were reviewed and approved by Ethical Committee of the School of Economics and Management, Beijing Jiaotong University. Written informed consent for participation was not required for this study in accordance with the national legislation and the institutional requirements.

## Author contributions

XZ and RZ contributed to this study, conceived and designed the study, developed the research model, designed the questionnaire, and drafted the manuscript. XZ mainly conducted data collection and analysis. RZ modified the manuscript. Both authors approved the final version of the manuscript for submission.

## Funding

This work was supported by a major project of the National Social Science Foundation of China (Grant Number 18ZDA086), a project of the National Natural Science Foundation of China (Grant Number 62173025), and a key project of the Beijing Social Science Foundation Research Base (Grant Number 18JDGLA017). Meanwhile, this work was supported by Beijing Logistics Informatics Research Base.

## Conflict of interest

The authors declare that the research was conducted in the absence of any commercial or financial relationships that could be construed as a potential conflict of interest.

## Publisher's note

All claims expressed in this article are solely those of the authors and do not necessarily represent those of their affiliated organizations, or those of the publisher, the editors and the reviewers. Any product that may be evaluated in this article, or claim that may be made by its manufacturer, is not guaranteed or endorsed by the publisher.
