# Supplementary material for: Factors influencing patients' opt-in intention of exchanging health information
Source: Front Public Health. 2022 Oct 17;10:907141. doi: 10.3389/fpubh.2022.907141 (PMC9645240; doi:10.3389/fpubh.2022.907141)
Supplement: Supplementary file 1 [file Table_1.DOCX]

Supplementary Material

1. Have you ever visited a health care provider participating in a health information exchange(HIE) network?

- Yes - No

2. Please specify the main reason that made you become aware of HIE projects:

- -  I have become aware of HIE through visiting a doctor who participates in an HIE network
- -  I have become aware of HIE through participating in an HIE network (By using Personal Health Record systems)
- -  I have become aware of HIE through the internet searching and social media (online information gathering)
- -  I have become aware of HIE through reading magazines/ newspaper articles or ads
- -  I have become aware of HIE through my friends and family
- -  Other (Please specify)

**Table A1**. Measurement Instruments.

| **Construct** | **Scale/Scoring** | **Items** |
| --- | --- | --- |
| Opt-in intention | - 7-point Likert - Strongly disagree to strongly agree | 1. I intend to use HIE in the next 12 months. |
|  |  | 2. I predict I would use HIE in the next 12 months. |
|  |  | 3. I plan to use HIE in the next 12 months. |
| Information sensitivity | - 7-point Likert - Strongly disagree to strongly agree | **Generally, I believe that information about _____ is sensitive.** |
|  |  | 1. Medication. |
|  |  | 2. State of my health at present. |
|  |  | 3. Fitness at present. |
|  |  | 4. Medical history. |
|  |  | 5. Addictions. |
| Perceived transparency of HIE | - 7-point Likert - Strongly disagree to strongly agree | 1. The HIE has the notice that clearly states type of health data collected and shared. |
|  |  | 2.The HIE has the notice that clearly states the purposes for which the health data is to be exchanged |
|  |  | 3. The HIE has the notice that clearly identifies any potential recipients of the data. |
|  |  | 4. The HIE has the notice that clearly explains how the shared personal information will be used. |
|  |  | 5. The HIE has the notice that clearly states whether the exchange of the requested data is voluntary or required. |
| Perceived behavior control | - 7-point Likert - Strongly disagree to strongly agree | 1. I am able to apply the HIE. |
|  |  | 2. I do not have adequate knowledge to use HIE. |
|  |  | 3. I have adequate resources to use the HIE. |
|  |  | 4. Using HIE is entirely within my control. |
| Trust in HIE | - 7-point Likert - Strongly disagree to strongly agree | 1. Even if not monitored, I would trust HIE to do the job right. |
|  |  | 2. I trust HIE that protects personal information. |
|  |  | 3. I believe that HIE is trustworthy. |
| Health status | - 7-point Likert - Strongly disagree to strongly agree | 1. I experience major pains and discomfort for extended period of time. |
|  |  | 2. When it comes to chronic condition(s), I believe that my condition is excellent. |
|  |  | 3. In general I believe that the state of my health is very good. |

**
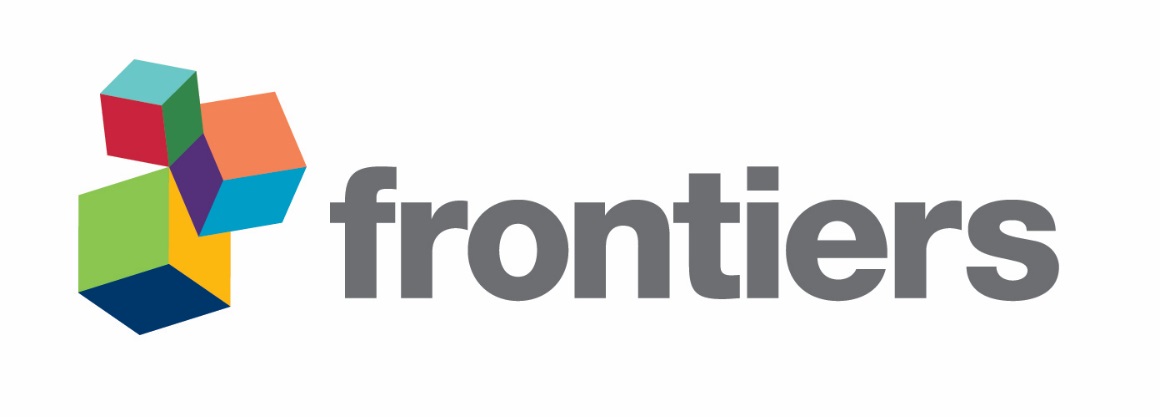
**
